# Genotypic and Phenotypic Characterization of *Salmonella* Typhimurium Strains Isolated from Swine in the Southern Region of Brazil

**DOI:** 10.1007/s00284-026-04762-z

**Published:** 2026-02-21

**Authors:** Giovana do Nascimento Pereira, Isabella Cardeal Campos, Carolina Nogueira Gomes, Felipe Pinheiro Vilela, Jalusa Deon Kich, Marc William Allard, Juliana Pfrimer Falcão

**Affiliations:** 1 Faculdade de Ciências Farmacêuticas de Ribeirão Preto, Universidade de São Paulo, Ribeirão Preto, SP Brasil; 2 Faculdade de Ciências Agrárias e Veterinárias – Universidade Estadual Paulista, Jaboticabal, SP Brasil; 3 Empresa Brasileira de Pesquisa Agropecuária-Suínos e Aves-EMBRAPA, Concórdia, SC Brasil; 4 Food and Drug Administration-FDA, College Park, MD USA

## Abstract

*Salmonella* Typhimurium has long been one of the most frequently isolated serovars in animal and human infections. Pork has been involved in the dissemination of *S.* Typhimurium to humans and plays an important role in the epidemiology of this infection. This study aimed to characterize genotypically and phenotypically *S.* Typhimurium strains isolated from swine in Brazil. The genomic relatedness among 17 of the 18 S. Typhimurium genomes was ≥ 90% according to Gegenees analysis, while ANI analysis showed ≥ 98.2% similarity across all 18 genomes, with most strains belonging to SNP cluster PDS000201117.2. Virulence factors and stress-related genes were searched at NCBI Pathogen Detection. All strains carried the *iroB*,* iroC*,* sinH*,* asr*,* golS*, and *golT* genes. Under acid stress, all strains survived after 10 min and 1 h. Under oxidative stress, 17 (94.44%) strains survived after 10 min, and 11 (61.11%) strains survived after 1 h. The invasion rates in Caco-2 cells ranged from 37.50% to 100%, and survival in the macrophage assay ranged from 37.50% to 87.50%. In conclusion, the close genetic relationship observed among the *S*. Typhimurium strains isolated from swine studied may suggest that a predominant subtype may have prevailed in Brazil’s swine sources. The high prevalence of some heavy metal tolerance encoding genes is alarming due to their potential to influence in the co-selection of *S.* Typhimurium strains resistant to antibiotics. Moreover, the presence of some virulence genes and the notable stress survival and cell invasion capacities highlighted the importance of surveillance to prevent the dissemination through food of virulent *S.* Typhimurium strains present in livestock to humans.

**Supplementary Information:**

The online version contains supplementary material available at 10.1007/s00284-026-04762-z.

## Introduction

Salmonellosis is one of the main causes of foodborne diarrheal diseases worldwide [1]. According to the CDC’s estimations, *Salmonella* is responsible for approximately 1.35 million infections, 26,500 hospitalizations, and 420 deaths annually in the United States [2]. In the European Union, salmonellosis is ranked as the second most frequently reported zoonosis and emerged as the predominant cause of foodborne outbreaks over the past decade [3–5] In Brazil, this bacterium has been reported as the third most common microorganism related to water and foodborne outbreaks [6].

*Salmonella enterica* subspecies *enterica* serovar Typhimurium (*S.* Typhimurium) has long been one of the most frequently isolated serovars in animals and humans across various regions, including The United States, The European Union, Australia, Africa, and Brazil [7–10]. This serotype has a broad animal host range, infecting livestock, domestic fowl, rodents, and birds [11]. It is transmitted mainly through the consumption of contaminated water or food, such as pork and poultry meat, and undercooked or raw eggs [12, 13].

Pork has been strongly involved in the dissemination of *S*. Typhimurium to humans, and plays an important role in the epidemiology of this infection [14, 15]. According to the Brazilian Association of Animal Protein (ABPA), Brazil is the fourth-largest producer and exporter of pork globally. In 2023, 5.156 million tons were produced, and 1.230 million tons were exported [16]. In addition, Brazil retains its global leadership in the cost of production, measured in dollars per kilogram of live swine [17].

The pathogenesis of *S.* Typhimurium is promoted by multiple virulence genes, that can be presented in the chromosome, plasmids, integrated bacteriophage DNA, *Salmonella* pathogenicity islands (SPIs), and *Salmonella* genomic islands (SGIs) [18]. In addition, the expression of these virulence genes is controlled by multiple regulators arranged within intricate regulatory networks, which in turn respond to a diverse range of environmental signals [19].

Throughout the infection process, *S*. Typhimurium may face diverse stress factors, such as changes in temperature, the action of antimicrobial peptides (AMPs), low pH, bile salts, nutrient scarcity, reactive oxygen species (ROS), and nitrosative stress [20]. These hostile environments trigger the expression of virulence genes and activate a set of stress response genes that influence the pathogen’s capacity to resist challenging conditions [20].

Different studies have investigated the survival capacity of foodborne bacteria under challenging conditions, including survival in acid and oxidative stress [21–23]. Additionally, the assessment of *Salmonella* invasion in colorectal adenocarcinoma cells (Caco-2) and its survival in U-937 human macrophages has demonstrated to be a valuable method for characterizing and differentiating strains in terms of their virulence and survival [22, 24, 25].

Regarding antimicrobial resistance, antibiotics have been widely employed both for treating animal diseases and as growth-promoting agents [26]. However, in some countries, including the European Union and Brazil, the use of antibiotics as growth promoters has been banned due to food safety and health issues [6, 26]. On the other hand, metals such as copper, zinc, cobalt, chromium, and manganese are commonly found in nature, and some are also considered environmental contaminants due to human pollution [26]. Additionally, they are found to be extensively used in animal feed because of their growth-promoting and antimicrobial properties. However, their use may contribute to antibiotic resistance through co-selection, where the association arises from the co-location of resistance genes, shared resistance mechanisms, or co-regulation of resistance pathways [26].

Therefore, alongside the study of virulence genes, the detection of heavy metal resistance genes is crucial in zoonotic bacteria, including *S*. Typhimurium. In this context, whole genome sequencing (WGS) has proven to be a powerful tool not only for identifying these genes but also for assessing the phylogenetic relationships between different strains of this genus [27, 28].

In this way, this study aimed to verify the genomic correlation, presence of virulence, heavy metal tolerance, and stress-related genes through WGS for *S.* Typhimurium isolates from swine in the Southern region of Brazil. Additionally, the ability to survive acid and oxidative stresses, invade Caco-2 cells, and survive in human macrophages were also evaluated for these strains.

## Materials and Methods

### Bacterial Strains

A total of 18 *Salmonella* Typhimurium strains isolated from swine in the Santa Catarina state, located in the Southern Region of Brazil, were studied (Table 1). These strains were systematically selected from the Brazilian Agricultural Research Corporation (EMBRAPA) collections to represent isolates from sporadic cases that occurred from 2000 to 2012 in Santa Catarina. The strains were cryopreserved at −80 °C.

**Table 1:** CFSAN and designation of strains, year of isolation, source, and SNP cluster of the 18 *Salmonella* typhimurium strains isolated from swine between 2000 and 2012 studied

CFSAN Number	Designation of Strains	Year	Source	SNP Cluster
CFSAN068033	STM 739	2000	Mesenteric lymph node	-
CFSAN068028	STM 22	2004	Inguinal lymph node	PDS000099352.1
CFSAN068029	STM 58	2004	Swine feces	PDS000201117.2
CFSAN068040	STM 1212	2005	Mesenteric lymph node	-
CFSAN068043	STM 1218	2005	Mesenteric lymph node	PDS000201128.4
CFSAN068044	STM 1220	2005	Mesenteric lymph node	-
CFSAN068045	STM 1221	2005	Mesenteric lymph node	PDS000201117.2
CFSAN068046	STM 1222	2005	Mesenteric lymph node	PDS000201117.2
CFSAN068047	STM 1224	2005	Mesenteric lymph node	PDS000201117.2
CFSAN068031	STM 343	2006	Herd environment	PDS000201117.2
CFSAN068042	STM 1214	2006	Herd environment	-
CFSAN068041	STM 1213	2007	Herd environment	PDS000201117.2
CFSAN068030	STM 338	2008	Swine feces	PDS000201117.2
CFSAN068032	STM 345	2008	Swine feces	PDS000201117.2
CFSAN068034	STM 812	2011	Swine urine	PDS000027042.1
CFSAN068035	STM 824	2011	Swine urine	PDS000027042.1
CFSAN068036	STM 1206	2011	Swab drag	PDS000201128.4
CFSAN068037	STM 1207	2012	Swab carcass	PDS000027047.16

### Genomic Characterization

The DNA extraction, genome sequencing, and the accession numbers of the strains studied were previously reported by Seribelli et al. [27]. The draft genomes of the 18 *S*. Typhimurium strains were analyzed to determine their genomic relatedness and virulence potential.

The presence of virulence factors and stress-related genes, such as those related to tolerance against acid, biocide, and heavy metals, were searched using the filters “Virulence genotypes” and “Stress genotype”, respectively, at NCBI Pathogen Detection (https://www.ncbi.nlm.nih.gov/pathogens/isolates/). The search for these genes was performed by searching the accession numbers of the 18 strains analyzed (Table 1).

The genomic relatedness and similarity among the 18 strains studied were assessed using two complementary approaches. First, an analysis of fragmented genomes was performed with Gegenees 3.1, as previously described [29]. Briefly, draft genomes were fragmented using a 500 bp fragmentation length and step size, were aligned and compared using the BLASTn method, and similarity scores were calculated. The resulting similarity matrix was exported in the *nexus* extension and used in the SplitsTree4 software [30] to create a phylogenetic tree based on the Neighbor-Joining method.

In addition, Average Nucleotide Identity (ANI) analysis was conducted to provide a quantitative measure of overall genome similarity among the strains. ANI values were calculated using FastANI Calculator (https://gtdb.ecogenomic.org/tools/fastani), and pairwise percentages were used to confirm species-level identity and to further evaluate the genetic relatedness of the isolates, following approaches similar to those described [31] for high-throughput genome comparisons.

The SNP Cluster analysis of NCBI Pathogen Detection was employed to analyze the genomic correlation of the 18 strains studied to additional global genomes (https://www.ncbi.nlm.nih.gov/pathogens/isolates/). SNP Cluster was also searched based on the accession number of the 18 strains analyzed (Table 1).

### Acid and Oxidative Stress Survival

Acid and oxidative stress survival was assessed for all 18 studied strains. The virulent *S.* Typhimurium strain ATCC 14,028 was used as a control in the stress experiments. Survival under acid and oxidative stresses was assessed according to Fang et al. [32] with modifications.

The *S.* Typhimurium strains were grown in Luria Bertani (LB) medium until they reached the stationary growth phase (16 h). The optical density (OD) was set to an OD_600_ of 0.2, equivalent to approximately 1 × 10^8^ colony-forming units (CFU) per ml [33], and then 1 mL was removed and centrifuged at 8,000 × g for 5 min.

For the acid stress tolerance assay, the pellets were resuspended in 1 mL of sodium citrate buffer 100 mM pH7.0 (control) and sodium citrate buffer 100 mM pH4.5 (stress). For the oxidative stress tolerance assay, the pellets were resuspended in 1 mL of saline 0.8% (control) and saline 0.8% supplemented with H_2_O_2_ 15mM (stress). Control and stress aliquots were taken after 10 min and 1 h. In both tests, the suspensions were serially diluted (10^–1^–10^–6^) and plated on LB agar.

The impact of stress was assessed by calculating the surviving fraction, which is determined by dividing the number of stressed colonies by the number of control colonies. The experiments were carried out in biological triplicate. Comparisons between the means of the treatments referring to the tolerance tests were performed using Student’s t-test with α = 5% significance level.

### Caco-2 Invasion Assay

The ability to invade human colon adenocarcinoma cells (Caco-2) was compared between the 18 *S*. Typhimurium isolates (Table 1) and *S*. Typhimurium ATCC 14,028 according to the protocol described by Fierer et al. [34, 35], Moreira et al. [35], and Pfeifer et al. [36] with some modifications.

Briefly, Caco-2 epithelial cells were grown in Dulbecco’s modified Eagle medium (DMEM) (Sigma-Aldrich, Arklow, Ireland) supplemented with 10% fetal bovine serum (Life Technologies, CA, USA) and 1% antibiotic/antimycotic (Life Technologies) in 75-cm^2^ tissue culture flasks at 37 ℃ in a 5% CO_2_ atmosphere until cell layers were confluent. The cells were seeded into 12-well tissue culture plates at a concentration of 1 × 10^5^ cells per well and plates were incubated in a 5% CO_2_ atmosphere at 37 ℃ for 12 days to provide the polarization of the cells [37].

The *S*. Typhimurium strains were grown in Luria–Bertani (LB) medium at 37 °C until the early stationary phase (16 h). The optical density (OD) was adjusted to an OD_600_ = 0.2, and then 1 mL was removed and centrifuged at 8000×g for 5 min. The cell pellet was resuspended in 1 mL of DMEM cell culture medium without antibiotics and fetal bovine serum.

The strains were added to Caco-2 monolayers with a multiplicity of infection (MOI) ratio of 100:1 (bacterial/epithelial cell). Bacterium-cell interactions occurred over 90 min in a 5% CO_2_ atmosphere at 37 °C [37]. After incubation, the wells were washed with phosphate-buffered saline (PBS 1X) and then treated with 1 mL of DMEM containing 30 µg/mL of gentamicin. Plates were then incubated for an additional 90 min. Subsequently, 1 mL of DMEM without antibiotics and fetal bovine serum was added to each well, which were incubated for 3 h in a 5% CO_2_ atmosphere at 37 °C. The cells were washed with PBS 1X, followed by cell lysis using a 1% Triton X-100 solution for 5 min.

The CFU/mL was determined by serial dilutions and plating onto LB agar medium plates. Incubation occurred over 18–24 h at 37 °C. Post-incubation the CFU were counted. The experiments were conducted in biological triplicate, and all plates included a negative control consisting only of cells in the wells.

The determination of invasion percentages in Caco-2 cells involved the division of the Log CFU/mL value post-cell lysis by the logarithm of the initial inoculum (log 10^7^), with subsequent multiplication by 100.

### Survival Assay in U-937 Human Macrophages

The monocytes were cultured in Roswell Park Memorial Institute Medium (RPMI) (Life Technologies), supplemented with 10% bovine fetal serum (Life Technologies), in a 5% CO2 atmosphere at 37 °C until confluent. The cells were seeded into 24-well tissue culture plates at a concentration of 1 × 10^5^ cells per well. To induce the differentiation of monocytes into macrophages, the medium was supplemented with 10 nM phorbol myristate acetate (PMA; Sigma), and the plates were incubated in a 5% CO_2_ atmosphere at 37 °C for 24 h. After 24 h, the medium was replaced, and the plates were subjected to an additional 24-hour incubation without the PMA presence.

The 18 *S.* Typhimurium strains were grown in Luria Bertani (LB) medium until reaching the stationary growth phase (16 h) as described in the Caco-2 invasion assay and 1 mL aliquots were centrifugated at 8,000×g for 5 min. The formed pellet was washed with PBS 1X three times, and then the bacteria cells were opsonized with 20% of mouse serum (Sigma-Aldrich) at 37 °C for 15 min. Following this step, cells were centrifugated at 8000×g for 5 min and subsequently resuspended in 1 mL of RPMI cell culture medium, free from antibiotics and/or fetal bovine serum.

For the infection of U-937 cells with *S*. Typhimurium, a MOI of 100:1 was used. Bacterium-cell interactions were allowed to unfold over 30 min in a 5% CO_2_ atmosphere at 37 °C, by the protocol described by Moreira et al. [36]. and Detweiler et al. [38] with modifications. After the incubation period, the wells were washed with PBS 1X and were treated with 1 mL of RPMI containing 30 µg/mL of gentamicin.

The plates were incubated for additional 90 min. After incubation, a PBS 1X wash was carried out, and each well received 1 mL of cell medium with no antibiotics and bovine fetal serum and incubated for 3 h in a 5% CO_2_ atmosphere at 37 °C incubation as previously described by Moreira et al. [36]. The cells were washed with PBS 1X followed by a cell lysis with a 1% Triton X-100 solution for 5 min at room temperature.

Serial dilutions were performed and plated onto LB agar medium plates. The incubation, the colony-forming units counting as well as the survival percentage in U-937 human macrophages were determined according to what is described in “Caco-2 invasion assay”. The experiments were conducted in biological triplicate, and all plates included a negative control consisting only of cells in the wells.

### Statistical Analysis

In the phenotypic assays, Student’s t-test was performed in Microsoft Excel^®^ to compare the survival of individual *S.* Typhimurium strains with the reference strain *S.* Typhimurium ATCC 14,028. The test was also applied to assess the association between the presence or absence of stress-related genes and the survival of *S.* Typhimurium under the different phenotypic conditions.

## Results

### Genomic Analyses

All the 18 *S.* Typhimurium genomes analyzed harbored genes *iroB* and *iroC*, related to the production of siderophores, *sinH*, that encodes an intimin-like inverse auto transporter, *asr*, related to acid resistance, and *golS* and *golT*, related to gold tolerance. The operons encoding copper and silver tolerance genes (*pcoABCDERS* and *silABCEFPRS*, respectively) were detected in nine strains (50%). Genes *qacEdelta1* and *qacL*, related to resistance against quaternary ammonium compounds, were found in five (27.7%) and four (22.2%) strains, respectively. Tellurium tolerance genes *terDWZ* were detected in five strains (27.7%). Mercury tolerance genes *merR* and *merT* were detected in four (22.2%) strains, *merC* and *merP* in three (1.6%), *merD* and *merE* in two (1.1%), and *merA* in a single strain (0.5%). Gene *cdtB*, encoding a cytolethal distending toxin, was detected in a single strain (0.5%).

**Fig. 1:**
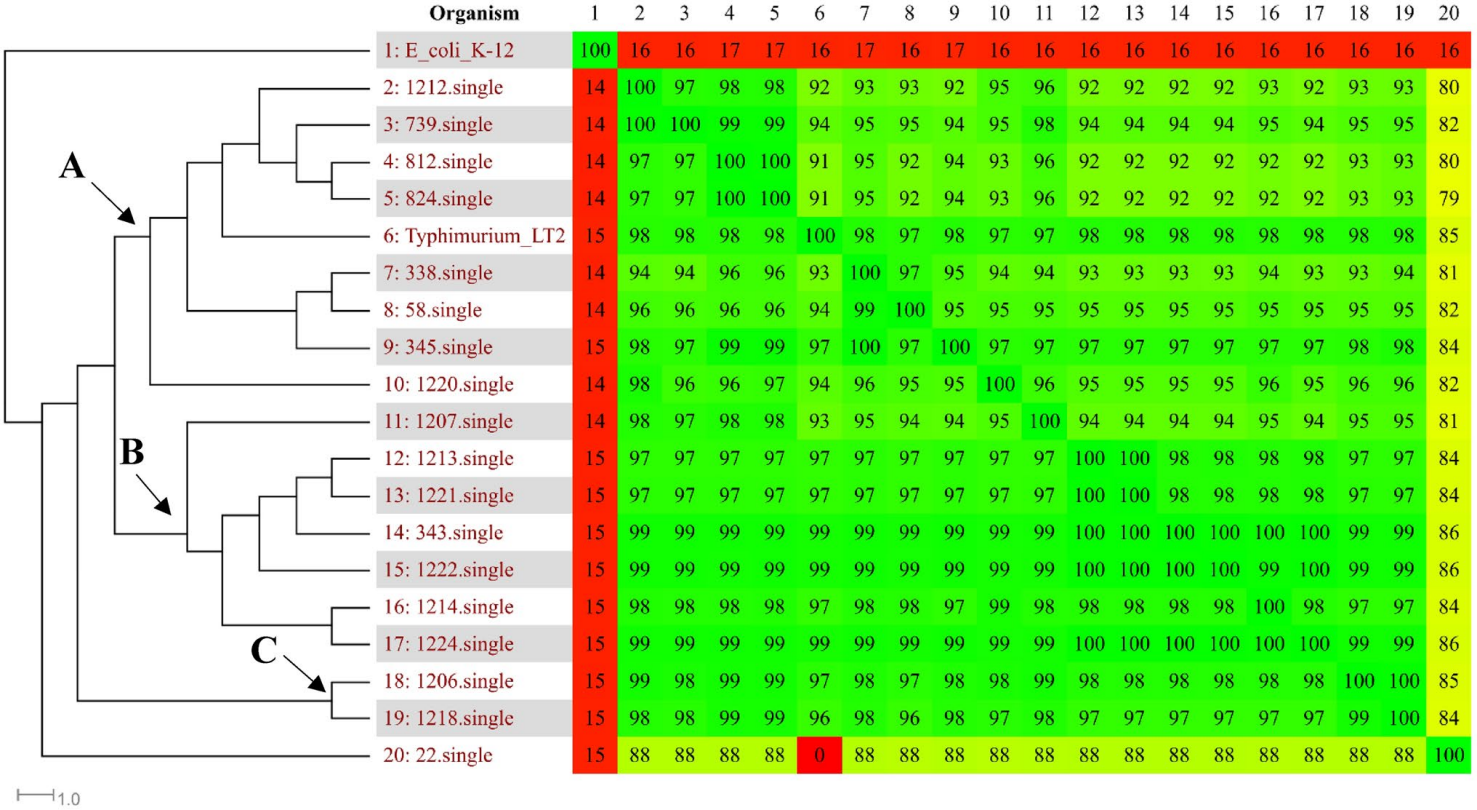
Phylogenomic tree and heatmap analyses of the18 *Salmonella* Typhimurium strains studied isolated from swine in Brazil. Comparisons between the variable content of all the strains were plotted as percentages of similarity on the heatmap using Gegenees. The heat map values indicate the percentage of similarity between the genomes; red: lower similarity and green: higher similarity. The percentage of similarity was used to generate a phylogenomic tree with SplitsTree

In the phylogenomic analysis conducted through Gegenees, a similarity percentage ≥ 79% was detected among all the 18 genomes analyzed, with 17 (94.44%) of the strains presenting a similarity ≥ 90%, suggesting a close genomic relatedness among the majority of *S.* Typhimurium strains studied (Fig. 1).

The *nexus* file exported from the Gegenees software was subsequently used in SplitsTree4 to generate a phylogenetic tree, grouping *S.* Typhimurium into three clusters named A, B, and C. Cluster A comprised eight strains isolated from mesenteric lymph nodes, swine feces, and swine urine between 2000 and 2011. Cluster B included seven strains isolated from mesenteric lymph nodes, herd environments, and swine carcasses between 2005 and 2012. Finally, cluster C contained two strains isolated from mesenteric lymph nodes and swab drags in 2005 and 2011. The strain STM 22, isolated from an inguinal lymph node, was not grouped into any of the formed clusters.

**Fig. 2:**
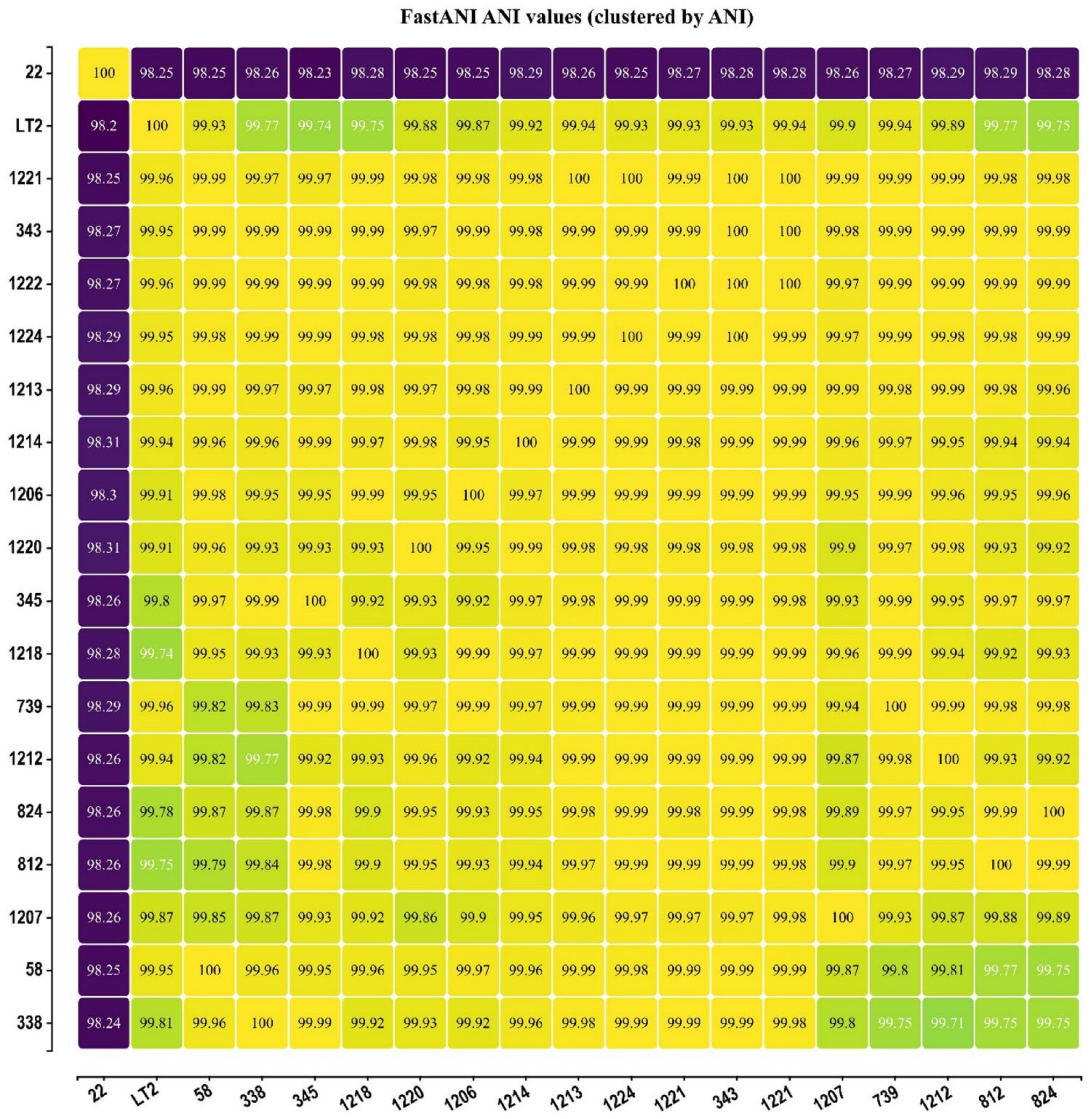
Average Nucleotide Identity (ANI) analysis among 18 *Salmonella* Typhimurium strains studied isolated from swine in Brazil. ANI values were calculated using the FastANI Calculator

The Average Nucleotide Identity (ANI) values obtained with FastANI revealed a high degree of genomic similarity among the 18 *S.* Typhimurium strains analyzed (Fig. 2). Pairwise ANI values ranged from 98.2% to 100%, with most comparisons showing values above 99.9%, confirming the species-level identity of all isolates.

The analysis of SNP clusters showed that 14 of the 18 genomes (77.7%) were assigned to five specific groups, while four genomes (22.2%) were not assigned to any group (last update: September 19th, 2025). SNP cluster PDS000201117.2 was assigned to 8 (44.4%) genomes studied and also harbored an additional 59 genomes of strains isolated from clinical and environmental/other sources between 2016 and 2024 in Brazil, Denmark, France, Paraguay, Lebanon, the United Kingdom, and the United States. SNP cluster PDS000027047.16 was assigned to one strain studied and 18 additional genomes of strains from Brazil and the United States isolated between 2016 and 2024 from clinical and environmental/other sources. SNP cluster PDS000201128.4 was assigned to two strains studied and 31 additional genomes of strains from Brazil, Denmark, Paraguay, the United Kingdom, Portugal, and the United States isolated between 2015 and 2025 from clinical and environmental/other sources. SNP cluster PDS000099352.1 was formed by one strain from the present study and an additional genome from the United Kingdom of a strain from a clinical source isolated in 2021. Finally, SNP cluster PDS000027042.1 was formed exclusively by two strains analyzed in the present study.

### Acid Stress

**Fig. 3:**
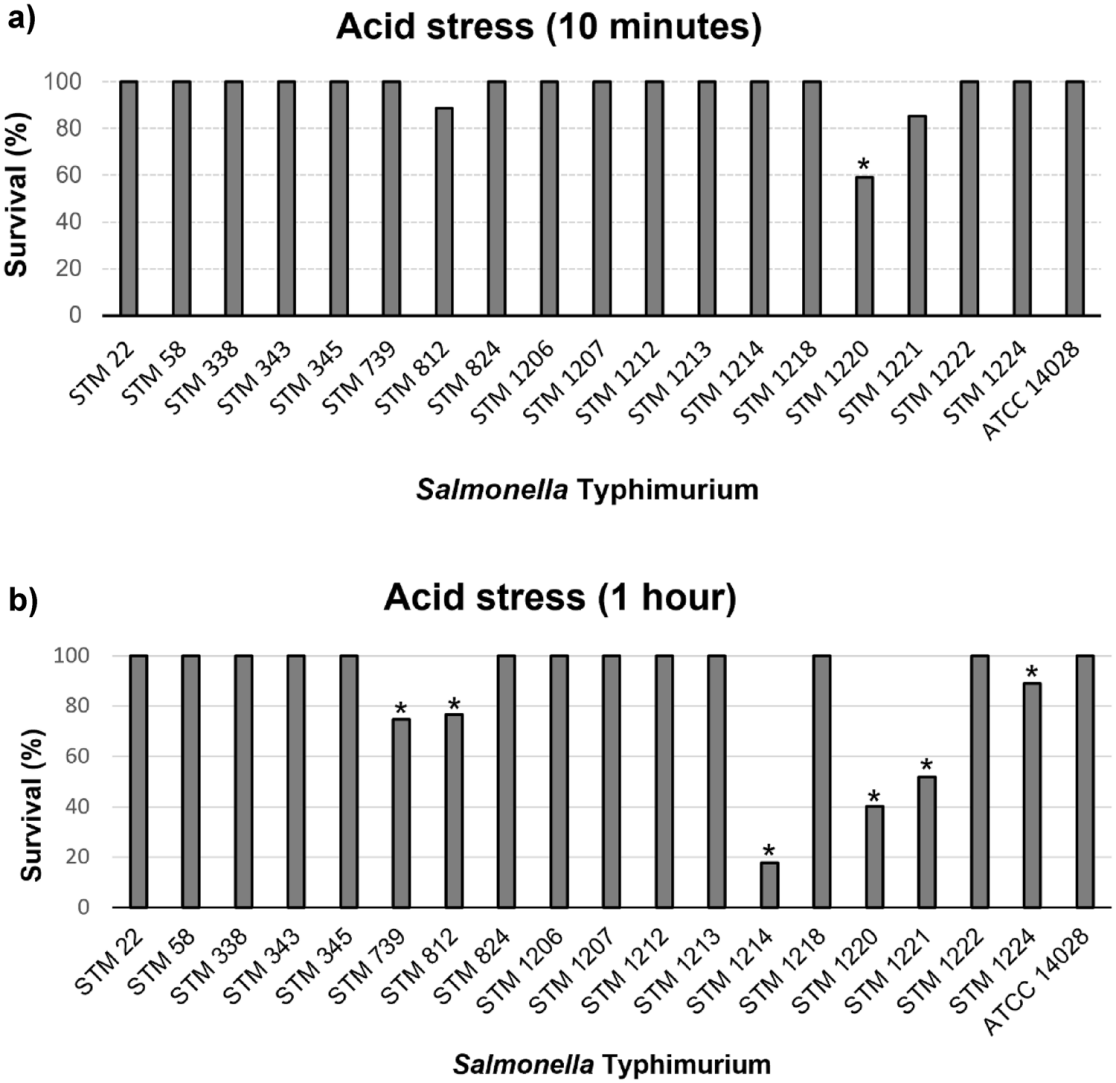
Survival of 18 *Salmonella* Typhimurium strains studied isolated from swine in Brazil after 10 min of acid stress exposure (**a**) and after 1 h of acid stress exposure (**b**). *P ≤ 0.05

All 18 *S*. Typhimurium strains isolated from swine survived under acid stress after 10 min and 1 h (Fig. 3a and b).

A total of 17 strains (94.44%) had approximate survival rates of 100% after 10 min of acid stress, as the highly virulent *S*. Typhimurium ATCC 14,028 strain. In addition, only strain STM 1220 presented a survival rate of 59.13% and survived significantly less than the ATCC 14,028 strain after 10 min of acid stress (Fig. 3a).

Furthermore, 12 strains (66.67%) had approximate survival rates of 100% after 1 h of acid stress, as the highly virulent *S*. Typhimurium ATCC 14,028 strain. In addition, 6 strains (33.33%; STM 739, STM 812, STM 1214, STM 1220, STM 1221, and STM 1224) presented a survival of around 17.67% to 88.89% and survived significantly less than the ATCC 14,028 strain after 1 h of acid stress (Fig. 3b).

### Oxidative Stress

**Fig. 4:**
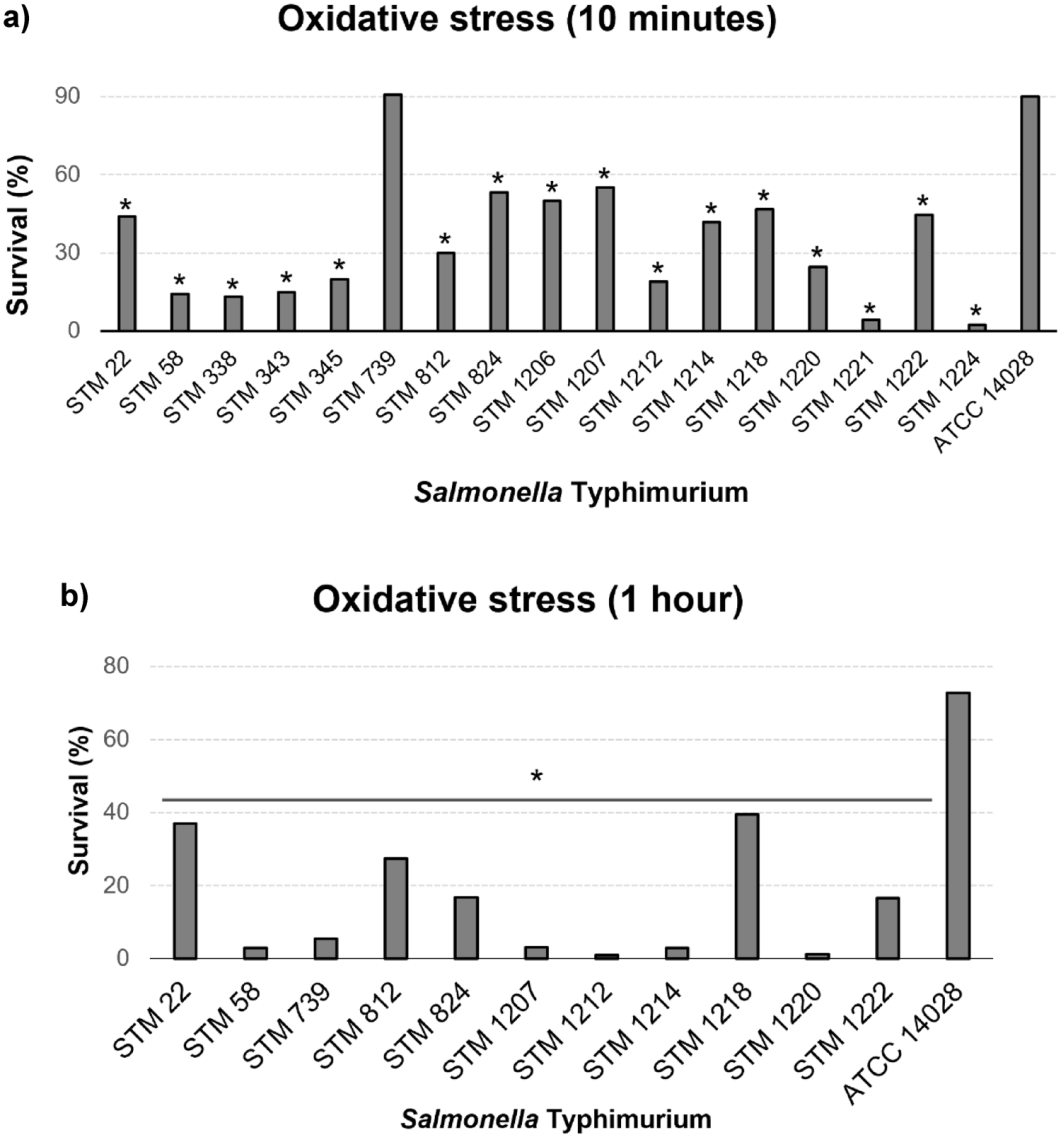
Survival of 18 *Salmonella* Typhimurium strains studied isolated from swine in Brazil after 10 min of oxidative stress exposure (**a**) and after 1 h of oxidative stress exposure (**b**). *P ≤ 0.05

The majority of the 18 *S*. Typhimurium strains isolated from swine survived oxidative stress after 10 min and 1 h (Fig. 4a and b).

After 10 min of oxidative stress, 17 strains (94.44%) survived. Only strain STM 739 presented a survival rate of 90.78% and survived similarly to ATCC 14,028 which presented a survival rate of 90.06%. Moreover, 16 strains (88.89%) had approximate survival rates of 2.30% to 55.07% and survived significantly less than ATCC 14,028 (Fig. 4a). The strain STM 1213 did not survive to 10 min of oxidative stress.

In addition, after 1 h of oxidative stress, 11 (61.11%) strains had approximate survival rates of 0.9% to 39.45% and survived significantly less than ATCC 14,028 (Fig. 4b). The strains STM 338, STM 343, STM 345, STM 1206, STM 1213, STM 1221, and STM 1224 did not survive to 1 h of oxidative stress.

### Caco-2 Invasion Assay

**Fig. 5:**
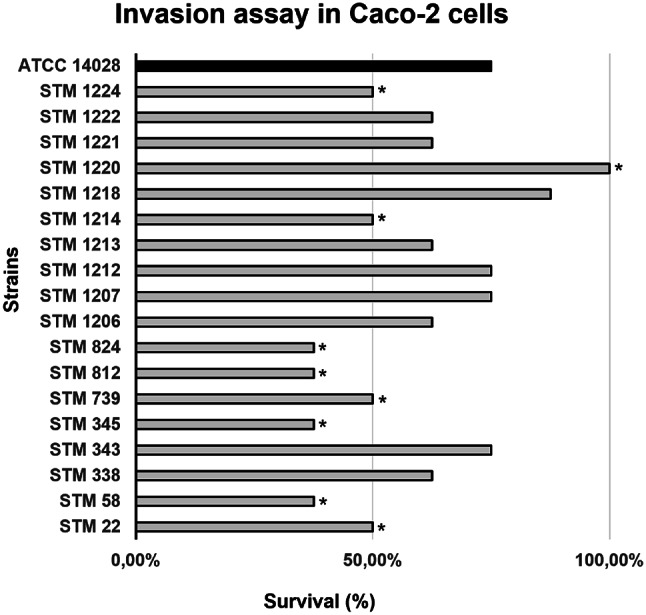
Invasion assay in Caco-2 epithelial cells for the 18 *Salmonella* Typhimurium strains studied isolated from swine in Brazil. *P ≤ 0.05

The invasion percentages in Caco-2 cells ranged from 37.50 to 100% and the *S.* Typhimurium 14,028 invasion percentage was 75% (Fig. 5). According to the Student’s t-test, only STM 1220 showed an invasion percentage significantly greater than the ATCC 14,028. In contrast, eight strains (44.44%; STM 22, STM 58, STM 345, STM 739, STM 812, STM 824, STM 1214, and STM 1224) invaded significantly less than the ATCC 14,028. Finally, nine strains (50%; STM 338, STM 343, STM 1206, STM 1207, STM 1212, STM 1213, STM 1218, STM 1221, and STM 1222) survived similarly to ATCC 14,028.

### Survival Assay in U937 Human Macrophages

**Fig. 6:**
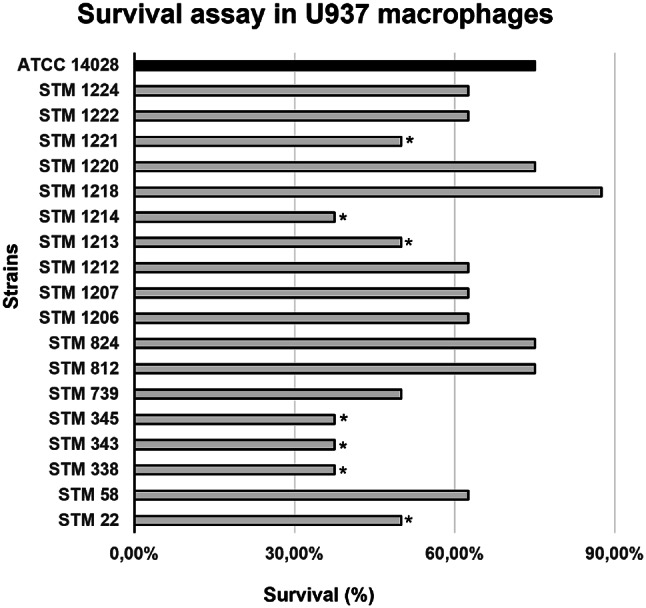
Survival assay in U937 human macrophages for the 18 *Salmonella* Typhimurium strains studied isolated from swine in Brazil. *P ≤ 0.05

The strains’ survival in the macrophage assay ranged from 37.50 to 87.50% and the ATCC 14,028 invasion percentage was 75% (Fig. 6). According to Student’s t-test, 11 strains (61.11%; STM 58, STM 739, STM 812, STM 824, STM 1206, STM 1207, STM 1212, STM 1218, STM 1220, STM 1222 and STM 1224) survived similarly to ATCC 14,028, and seven strains (38.9%; STM22, STM 338, STM 343, STM 345, STM 1213, STM 1214, and STM 1221) survived less than the ATCC 14,028.

### Association between Stress-Related Genes and Survival Rates

In general, no significant differences were observed between the presence or absence of a gene and survival rates under the tested stress conditions (*p *> 0.05). However, isolates carrying *qacL* exhibited significantly higher invasion of Caco-2 cells (*p *= 0.03), while the presence of *merCPRT* was associated with significantly increased survival under oxidative stress for 1 h (*p *= 0.03) (Table S1).

## Discussion

Understanding the genetic and phenotypic traits that contribute to the persistence and stress tolerance of *S.* Typhimurium strains isolated from swine environments provide valuable insights for surveillance and control strategies. These findings may support the development of targeted biosecurity measures and inform risk assessment practices within pork production systems.

Although this study is based on a limited number of isolates (*n* = 18) collected over 12 years ago, the studied strains provided a valuable historical perspective on the genetic diversity of *Salmonella*. Some of these strains belong to SNP clusters that continue to circulate in multiple countries, highlighting the long-term persistence and global dissemination of certain clones.

While these observations underscore the epidemiological relevance of the isolates, it is important to consider the temporal and geographic limitations of the dataset, as all isolates originated from swine in the Santa Catarina state, located in the Southern Region of Brazil, and represent sporadic and older samples. Therefore, the findings may not fully reflect the current *Salmonella* population in the region.

Nonetheless, this present study provided insights into the historical circulation of *Salmonella* clones, revealing aspects of their genetic content, including virulence factors and heavy metal resistance genes, as well as their ability to survive under acid and oxidative stress and to invade and persist in host cell lines, thereby enhancing our understanding of their epidemiology and adaptive potential in this key swine-producing region.

The pathogenicity of *Salmonella* is shaped by the cooperative action of genes to establish the infection [18]. The virulence genes detected using the NCBI Pathogen Detection tool were *iroBC* and *sinH* in all strains, and *cdtB* in a single strain (Table 2). The *iroBC* genes are related to siderophore production, while *sinH* encodes an intimin-like inverse autotransporter, and *cdtB* encodes a cytolethal-distending toxin.

**Table 2:** Virulence, metal resistance, and stress-related genes detected in the 18 *Salmonella* typhimurium studied strains

Strains	Virulence Genotypes	Acid Resistance	Biocide	Gold Tolerance	Mercury Tolerance	Copper Tolerance	Tellurium Tolerance	Silver Tolerance
STM 22	*iroBC*,* sinH*	*asr*		*golST*				
STM 58	*iroBC*,* sinH*	*asr*		*golST*	*merRT*	*pcoABCDERS*		*silABCEFPRS*
STM 338	*iroBC*,* sinH*	*asr*		*golST*	*merDE*			
STM 343	*iroBC*,* sinH*	*asr*		*golST*				
STM 345	*iroBC*,* sinH*	*asr*	*qacEdelta1*	*golST*	*merACDEPRT*	*pcoABCDERS*	*terDWZ*	*silABCEFPRS*
STM 739	*iroBC*,* sinH*	*asr*	*qacL*	*golST*				
STM 812	*iroBC*,* sinH*	*asr*	*qacEdelta1*	*golST*		*pcoABCDERS*		*silABCEFPRS*
STM 824	*cdtB*,* iroBC*,* sinH*	*asr*		*golST*				
STM 1206	*iroBC*,* sinH*	*asr*		*golST*				
STM 1207	*iroBC*,* sinH*	*asr*		*golST*				
STM 1212	*iroBC*,* sinH*	*asr*		*golST*	*merCPRT*	*pcoABCDERS*		*silABCEFPRS*
STM 1213	*iroBC*,* sinH*	*asr*		*golST*				
STM 1214	*iroBC*,* sinH*	*asr*		*golST*	*merCPRT*	*pcoABCDERS*	*terDWZ*	*silABCEFPRS*
STM 1218	*iroBC*,* sinH*	*asr*	*qacEdelta1*,* qacL*	*golST*		*pcoABCDERS*	*terDWZ*	*silABCEFPRS*
STM 1220	*iroBC*,* sinH*	*asr*		*golST*		*pcoABCDERS*	*terDWZ*	*silABCEFPRS*
STM 1221	*iroBC*,* sinH*	*asr*	*qacEdelta1*,* qacL*	*golST*		*pcoABCDERS*		*silABCEFPRS*
STM 1222	*iroBC*,* sinH*	*asr*	*qacEdelta1*,* qacL*	*golST*		*pcoABCDERS*	*terDWZ*	*silABCEFPRS*
STM 1224	*iroBC*,* sinH*	*asr*		*golST*				

It is important to note that the comprehensive virulence profile of these same strains has already been characterized by Seribelli et al. [27] using the Virulence Factors Database (VFDB), which identified 100 genes related to invasion, survival, colonization, fimbriae, and flagella production, as well as the presence of *Salmonella* Pathogenicity Islands (SPIs). Almeida et al. [39] also reported the presence of genes such as *sipA*,* sipD*,* flgK*,* flgL*,* fljB*,* invA*,* sopB*, and *sopE2* in all strains, and *sopD*,* ssaR*, and *sifA* in a subset of strains, using PCR-based analysis.

Therefore, instead of re-running VFDB, we focused on reporting the virulence genes identified via NCBI Pathogen Detection and comparing them with previously published datasets. Observed differences likely stem from variations in database content and detection platforms rather than genuine biological absence, underscoring the importance of integrating multiple data sources to interpret virulence potential accurately.

In the present study, we also detected the stress response genes *asr*,* golS*, and *golT* in all strains (Table 2). These stress response genes enable *S.* Typhimurium to survive in hostile environments. Specifically, the *asr* gene encodes an acid shock protein that allows for survival in acidic conditions, for example, inside host macrophages [40, 41]. Notably, all strains carried this gene and exhibited significant survival under acid stress and in U937 cells, suggesting that *asr* may contribute to tolerance under these conditions.

Additionally, *golS* and *golT* are related to gold tolerance, where *golS* is responsible for regulating *golT* expression. This, in turn, allows *Salmonella* Typhimurium to identify the presence of gold salts in the environment and trigger the necessary resistance response [42]. Similarly to our study, these genes were detected in all 132 *S.* Typhimurium strains isolated from diseased livestock and companion animals, and in all 32 *S.* Typhimurium strains isolated from humans in The United States [43, 44].

Genes related to tolerance to copper (*pcoABCDERS*), silver (*silABCEFPRS*), tellurium (*terDWZ*), and mercury (*merRACDEPT*) were also detected (Table 2). Copper and silver are frequently employed for their disinfectant and preservative qualities, while mercury may be present as a contaminant in animal feed [26].

The *pcoABCDERS* is a plasmid-borne copper system originally discovered in an *Escherichia coli* strain isolated from copper-fed pig feces [45, 46]. This system is responsible for oxidizing Cu + to Cu2 + and pumping this compound out of the cell [46]. It comprises three periplasmic proteins (*pcoACE*), a two-component regulatory system (*pcoRS*), an inner membrane protein (*pcoD*), and an outer membrane transporter (*pcoB*) [47].

In our study, the *pco* operon was detected in 50% of the strains analyzed. In comparison, it was identified in 12.1% of *S*. Typhimurium strains isolated from animals, but not in any human clinical cases in The United States. In contrast, it was found in 27.3% of *Salmonella* spp. isolates obtained from selected poultry farms in China [43, 44, 48].

The operon *sil* (*silABCEFPRS*) encodes the SilE periplasmic protein with silver-binding capabilities, SilP and SilABC silver efflux pumps, and SilF and SilG chaperones. Additionally, the SilS and SilR two-component signal transduction pair, intricately regulated by this operon, further contributes to the sophisticated control mechanism in response to silver exposure [49–52].

Similarly to our observations, Zhao et al. [43], Souza et al. [44], and Jiang et al. [48] reported that the *sil* operon exhibited a prevalence rate comparable to that of the *pco* operon in their strains, suggesting that both metal resistance determinants may share similar distribution patterns in *S.* Typhimurium.

The *terDWZ* genes belong to the *ter* operon and they are associated with tellurite resistance [53, 54]. This operon creates a multi-subunit complex linked to the inner surface of the bacterial membrane [55, 56]. TerC, comprising transmembrane proteins, interacts with TerD, TerB, and various other proteins [56, 57]. TerW serves as the initial functional product of the ter operon, known for its specific binding to the operon’s promoter region [58].

In contrast to previous reports, our study demonstrated a higher prevalence (27.8%) of the *ter* operon in *S.* Typhimurium strains compared to those isolated in The United States and China [43, 44, 48].

The *mer* operon regulates mercury binding and resistance, with its detected genes being *merRACDEPT*. MerR acts as a metal-responsive regulator, while MerD serves as a coregulator [59, 60]. The genes *merC*,* merE*, *merP*, and *merT* comprise a transport system for delivering mercuric ions across bacterial cytoplasmic membranes [59, 61]. Additionally, the *merA* gene encodes a cytoplasmic mercuric reductase that reduces toxic Hg2 + ions [59, 60]. In comparison with our study, *S.* Typhimurium strains isolated in The United States showed a higher prevalence of the genes associated to the *mer* operon [43].

Finally, the genes *qacEdelta1* and *qacL* encode efflux pumps that provide resistance against quaternary ammonium compound (QAC) antiseptics, which are cationic surfactant compounds interacting with the cytoplasmic membrane of bacteria [62, 63]. It is important to note that QACs are used in swine production for cleaning and disinfecting environments due to their antimicrobial properties, which are effective against a broad range of pathogens, making them ideal for sanitizing surfaces, equipment, and facilities [64–66]. Therefore, the presence of genes encoding resistance mechanisms to these compounds is a cause for concern.

Although *qacEdelta1* is the most frequently reported biocide-associated gene, followed by *qacL* in *S.* Typhimurium, our study demonstrated a lower prevalence compared to *S.* Typhimurium strains isolated from animals in The United States between 2002 and 2003, but a higher prevalence than in strains isolated from humans in the same country between 2017 and 2020 [43, 44].

In this study, a fragmented genome analysis was conducted using the Gegenees software to explore the evolutionary relationships among the strains. Clusters generated by the SplitsTree4 software, based on Gegenees data, revealed a close genetic relationship among most of the strains (Fig. 1). Additionally, the analysis also demonstrated that strains isolated from a common source exhibit greater genetic similarity among themselves since all strains isolated from swine feces, urine, and the environment were grouped within the same cluster, however, strains isolated from mesenteric lymph nodes were distributed across all the formed clusters (Fig. 1).

In addition, the ANI analysis further confirmed the species-level identity of the studied isolates, with values well above the 95–96% threshold commonly used to delineate bacterial species [31]. The remarkably high similarity (> 99.7%) observed among the swine isolates demonstrated pronounced genomic homogeneity, suggesting the persistence of closely related strains circulating within swine sources in Southern Brazil.

The search for SNP clusters using NCBI’s Pathogen Detection revealed that the strains are distributed across different clusters. Notably, the clusters PDS000201117.2, PDS000027047.16, and PDS000201128.4 included a significant number of strains isolated in Brazil, indicating that the studied strains share genetic similarity with additional genomes from this country. However, genomes isolated from other countries were also identified within the same SNP clusters, underscoring the presence of an *S.* Typhimurium subtype distributed across multiple countries and associated with diverse contamination sources.

Additionally, the PDS000027047.16 cluster specifically includes one genome from this study and 16 additional genomes from Brazil, isolated from clinical sources and the environment. This cluster predominantly consists of genomes isolated in Brazil. This may suggest that similar genetically related strains have likely been circulating among various sources, including pigs, clinical samples, the environment, and other sources in Brazil.

In this study, all 18 *S*. Typhimurium strains survived under acid stress after 10 min and 1 h (Fig. 3a and b). This result suggested that these strains can survive in acidic environments, such as chemically compromised ponds, soil, in the presence of food preservatives and Organic acids (OAs), and in the gastrointestinal tract. Similar to our results, in the study by Pereira et al. [23], all 80 *S*. Typhimurium biphasic and monophasic variant strains isolated from humans and non-humans in Brazil survived under acid stress after 10 min and 1 h.

Organic acids (OAs) can be used to control *Salmonella* and promote growth in pigs by adding them to water and/or feed. These acids reduce pH levels to 3.8–4.2 [67]. Studies have demonstrated that organic acids effectively improve productivity and delay *Salmonella* spp. exposure in animal husbandry and slaughterhouses [67, 68]. Additionally, several decontamination techniques have been tested to reduce *Salmonella* spp. growth on carcasses and fresh meat, such as spraying them with diluted solutions of organic acids [67, 69–72].

However, this pathogen has evolved a variety of mechanisms to adapt to a low pH environment, which can hinder the effectiveness of this method for controlling microorganisms [73]. Therefore, different variables must be evaluated when using these compounds, including acid concentration, exposure time, temperature, the duration of carcass storage, and the combination with other antimicrobial agents to reduce the risks of pathogenic bacteria in the food chain [74].

In this study, in the presence of hydrogen peroxide, the survival percentage of 17 (94.44%) strains was less than 55% after 10 min of exposure (Fig. 4a). Furthermore, after 1 h of exposure, this survival percentage decreased further, with seven (38.89%) strains not surviving (Fig. 4b). Similar results were observed in the study by Pereira et al. (2023) [23] with 80 S. Typhimurium strains isolated from humans and non-human sources in Brazil from 1983 to 2020.

One crucial mechanism employed by phagocytes to eliminate *Salmonella* Typhimurium is the production of ROS, including hydrogen peroxide (H_2_O_2_) [72]. This compound can cause damage to DNA, proteins, and various other biological molecules within bacterial cells [75]. Additionally, oxidative disinfectants like H_2_O_2_ have been tested as disinfectants in veterinary environments [76].

In this study, oxidative stress was found to be more effective in killing the studied *S*. Typhimurium compared to acid stress, and the presence of H_2_O_2_ reduced the percentage of *S.* Typhimurium strains. This suggests that disinfectant products containing this compound could be an alternative for reducing the growth of *S.* Typhimurium in slaughterhouse environments. Similarly, the study by Maertens et al. [76] recommends the use of a formulation containing peracetic acid and hydrogen peroxide at a concentration of 0.5% (5 mL/L).

In vitro, intestinal monolayers such as Caco-2 cells exhibit significant resemblance to human ileum epithelial cells and have been used in studies of *Salmonella* pathogenesis in the human gut [77].

In the Caco-2 invasion assay in this study, 14 (77.78%) strains had an invasion percentage ≥ 50%, and nine (50%) strains showed an invasion rate similar to ATCC 14,028 (Fig. 5). This suggests that most of the studied strains have an increased invasion capacity. Additionally, the year of isolation was not correlated with the virulence profiles.

In the study by Seribelli et al. [25] with *S.* Typhimurium isolated from humans and food in Brazil, a similar number of strains invaded more (35%), equal (32.5%), and less (32.5%) than its reference strain SL 1344. Similarly, in this study, 50% and 44.44% of the strains invaded equal to and less than our reference strain, ATCC 14,028, respectively. However, only one strain survived longer than the highly virulent ATCC strain.

During *S.* Typhimurium pathogenesis, the bacteria is internalized by macrophages after crossing the epithelial barrier, using phagocytes as an environment for replication and transportation [78]. For intracellular replication, *S.* Typhimurium hides within a membrane-bound compartment called the *Salmonella*-containing vacuole (SCV), which prevents its fusion with terminal acidic lysosomes [79].

In this study, the U-937 macrophage survival assay revealed that all strains exhibited a survival rate of over 30%, with 11 strains (61.11%) showing survival rates similar to the highly virulent ATCC 14,028 strain (Fig. 6). This data showed that most of the studied strains have significant macrophage survival. In contrast, Seribelli et al. [25] demonstrated that the majority of *S.* Typhimurium strains (62.5%) isolated from humans and food survived significantly less than the reference strain SL 1344.

Notably, this study shows that the strains STM 1218, STM 824, and STM 812 exhibited both high survival in macrophages and significant survival in the presence of H₂O₂, highlighting the agreement between these tests.

Finally, the results obtained from the phenotypic assays indicate that most stress-related genes evaluated were not significantly associated with survival under the tested conditions. However, isolates carrying *qacL* exhibited increased invasion of Caco-2 cells, and those with *merCPRT* showed higher survival under oxidative stress.

Although these findings suggest a potential link between these genes and stress tolerance or host-cell interactions, such associations have not been previously reported in the literature. Therefore, these results should be interpreted with caution and warrant further investigation to determine whether these genes indeed play a functional role in stress adaptation or pathogenicity.

## Conclusion

In conclusion, the close genetic relatedness observed among the *S.* Typhimurium strains studied isolated from swine may suggest the predominance of an adapted subtype that has persisted in swine populations in the Southern Region of Brazil. The high prevalence of some heavy metal tolerance encoding genes is alarming due to their potential to influence in the co-selection of *S.* Typhimurium strains resistant to antibiotics. Moreover, the presence of some virulence genes and the notable stress survival and cell invasion capacities highlighted the importance of surveillance to prevent the dissemination through food of virulent *S.* Typhimurium strains present in livestock to humans.

## Supplementary Information

Below is the link to the electronic supplementary material.

Supplementary Material 1

## Data Availability

Please contact the authors Giovana do Nascimento Pereira or Juliana Pfrimer Falcão for data requests.

## References

[1] World Health Organization (WHO) (2018) Salmonella (non-typhoidal). World Health Organization. https://www.who.int/news-room/fact-sheets/detail/salmonella-(non-typhoidal). Accessed 13 September 2024

[2] Centers for Disease Control and Prevention (CDC) (2020) Salmonella. Centers for Disease Control and Prevention. https://www.cdc.gov/salmonella/index.html#:~:text=CDC%20estimates%20Salmonella%20bacteria%20cause,%2C%20fever%2C%20and%20stomach%20cramps. Accessed 13 September 2024

[3] Schirone M, Visciano P (2021) Trends of major foodborne outbreaks in the European union during the years 2015–2019. Hygiene 1:106–119. 10.3390/hygiene1030010

[4] Sarno E, Pezzutto D, Rossi M et al (2021) A review of significant European foodborne outbreaks in the last decade. J Food Prot 84:2059–2070. 10.4315/JFP-21-096 34197583 10.4315/JFP-21-096

[5] European Food Safety Authority (EFSA), European Centre for Disease Prevention and Control (ECDC) (2023) The European union one health 2022 zoonoses report. Eur Food Saf Auth 21. 10.2903/j.efsa.2023.8442 10.2903/j.efsa.2023.8442 PMC10714251 38089471

[6] Ministério da Agricultura e Pecuária (MAPA) (2018) Diário oficial Da União. Imprensa Nac 1:23. https://pesquisa.in.gov.br/imprensa/jsp/visualiza/index.jsp?data=19/12/2018&jornal=515&pagina=23&totalArquivos=197 Accessed: 13 September 2024

[7] Ferrari RG, Rosario DKA, Cunha-Neto A et al (2019) Worldwide epidemiology of Salmonella serovars in animal-based foods: A meta-analysis. Appl Environ Microbiol 85:14. 10.1128/AEM.00591-19 10.1128/AEM.00591-19 PMC6606869 31053586

[8] Simpson KMJ, Mor SM, Ward MP, Walsh MG (2019) Divergent geography of Salmonella Wangata and Salmonella typhimurium epidemiology in New South Wales, Australia. One Health 7. 10.1016/j.onehlt.2019.100092 10.1016/j.onehlt.2019.100092 PMC6475636 31016222

[9] Fernandes SA, Tavechio AT, Ghilardi ACR et al (2022) Salmonella enterica serotypes from human and nonhuman sources in Sao Paulo State, Brazil, 2004–2020. Rev Inst Med Trop Sao Paulo. 10.1590/S1678-9946202264066 36197427 10.1590/S1678-9946202264066 PMC9528755

[10] Van Puyvelde S, de Block T, Sridhar S et al (2023) A genomic appraisal of invasive Salmonella Typhimurium and associated antibiotic resistance in sub-Saharan Africa. Nat Commun. 10.1038/s41467-023-41152-6 37872141 10.1038/s41467-023-41152-6 PMC10593746

[11] Kuria JKN (2024) Salmonellosis in Food and Companion Animals and Its Public Health Importance. Salmonella - Perspectives for Low-Cost Prevention, Control and Treatment. IntechOpen. 10.5772/intechopen.109324

[12] Galán-Relaño, Sánchez-Carvajal JM, Gómez-Gascón L et al (2022) Phenotypic and genotypic antibiotic resistance patterns in Salmonella typhimurium and its monophasic variant from pigs in Southern Spain. Res Vet Sci 152:596–603. 10.1016/j.rvsc.2022.09.028 36201906 10.1016/j.rvsc.2022.09.028

[13] Montoro-Dasi L, Lorenzo-Rebenaque L, Marco-Fuertes A et al (2023) Holistic strategies to control Salmonella Infantis: an emerging challenge in the European broiler sector. Microorganisms. 10.3390/microorganisms11071765 37512937 10.3390/microorganisms11071765 PMC10386103

[14] Gomes VTM, Moreno LZ, Silva APS et al (2022) Characterization of Salmonella enterica contamination in pork and poultry meat from São Paulo/Brazil: serotypes, genotypes and antimicrobial resistance profiles. Pathogens. 10.3390/pathogens11030358 35335682 10.3390/pathogens11030358 PMC8951033

[15] Roasto M, Bonardi S, Mäesaar M et al (2023) Salmonella enterica prevalence, serotype diversity, antimicrobial resistance and control in the European pork production chain. Trends Food Sci Technol 131:210–219. 10.1016/j.tifs.2022.12.007

[16] Associação Brasileira de Proteína Animal (ABPA) (2024) Relatório anual 2024. Associação Brasileira de Proteína Animal. https://abpa-br.org/wp-content/uploads/2024/04/ABPA-Relatorio-Anual-2024_capa_frango.pdf. Accessed 13 September 2024

[17] Empresa Brasileira de Pesquisa Agropecuária (EMBRAPA) (2023) Brasil tem o menor custo de produção de suínos entre 17 países. EMBRAPA. https://www.embrapa.br/busca-de-noticias/-/noticia/84538523/brasil-tem-o-menor-custo-de-producao-de-suinos-entre-17-paises#:~:text=O%20Brasil%20mant%C3%A9m%20a%20lideran%C3%A7a,por%20quilo%20vivo%20de%20su%C3%ADno. Accessed 13 September 2024

[18] Mthembu TP, Zishiri OT, El Zowalaty ME (2019) Detection and molecular identification of Salmonella virulence genes in livestock production systems in South Africa. Pathogens. 10.3390/pathogens8030124 31405078 10.3390/pathogens8030124 PMC6789496

[19] Guerra PR, Liu G, Lemire S et al (2020) Polyamine depletion has global effects on stress and virulence gene expression and affects HilA translation in Salmonella enterica serovar typhimurium. Res Microbiol 171:143–152. 10.1016/j.resmic.2019.12.001 31991172 10.1016/j.resmic.2019.12.001

[20] Arunima A, Swain SK, Ray S et al (2020) RpoS-regulated SEN1538 gene promotes resistance to stress and influences Salmonella enterica serovar enteritidis virulence. Virulence 11:295–314. 10.1080/21505594.2020.1743540 32193977 10.1080/21505594.2020.1743540 PMC7161692

[21] Gomes CN, Passaglia J, Vilela FP et al (2018) High survival rates of Campylobacter coli under different stress conditions suggest that more rigorous food control measures might be needed in Brazil. Food Microbiol 73:327–333. 10.1016/j.fm.2018.02.014 29526220 10.1016/j.fm.2018.02.014

[22] Campioni F, Gomes CN, Rodrigues D et al (2021) Phenotypic analyses of Salmonella enterica serovar Enteritidis strains isolated in the pre-and post-epidemic period in Brazil. Braz J Microbiol 52:173–183. 10.1007/s42770-020-00392-0 33107010 10.1007/s42770-020-00392-0 PMC7966658

[23] Pereira G, Seribelli AA, Gomes CN et al (2023) Virulence potential of Salmonella 1,4, [5],12:i:- strains isolated during decades from different sources in the Southeast region of Brazil. Braz J Microbiol 54:2827–2843. 10.1007/s42770-023-01145-5 37817050 10.1007/s42770-023-01145-5 PMC10689702

[24] Vilela FP, Gomes CN, Paziani MH et al (2020) Virulence traits and expression of bstA, fliC and sopE2 in Salmonella Dublin strains isolated from humans and animals in Brazil. Infect Genet Evol. 10.1016/j.meegid.2020.104193 31931258 10.1016/j.meegid.2020.104193

[25] Seribelli AA, Cruz MF, Vilela FP et al (2020) Phenotypic and genotypic characterization of Salmonella Typhimurium isolates from humans and foods in Brazil. PLoS One. 10.1371/journal.pone.0237886 32810191 10.1371/journal.pone.0237886 PMC7437471

[26] Mustafa GR, Zhao K, He X et al (2021) Heavy metal resistance in Salmonella Typhimurium and its association with disinfectant and antibiotic resistance. Front Microbiol. 10.3389/fmicb.2021.702725 34421860 10.3389/fmicb.2021.702725 PMC8371916

[27] Seribelli AA, da Silva P, Frazão MR et al (2021) Phylogenetic relationship and genomic characterization of Salmonella Typhimurium strains isolated from swine in Brazil. Infect Genet Evol. 10.1016/j.meegid.2021.104977 34174480 10.1016/j.meegid.2021.104977

[28] Pornsukarom S, Van Vliet AHM, Thakur S (2018) Whole genome sequencing analysis of multiple Salmonella serovars provides insights into phylogenetic relatedness, antimicrobial resistance, and virulence markers across humans, food animals and agriculture environmental sources. BMC Genomics. 10.1186/s12864-018-5137-4 30400810 10.1186/s12864-018-5137-4 PMC6218967

[29] Ågren J, Sundström A, Håfström T, Segerman B (2012) Gegenees: fragmented alignment of multiple genomes for determining phylogenomic distances and genetic signatures unique for specified target groups. PLoS One. 10.1371/journal.pone.0039107 22723939 10.1371/journal.pone.0039107 PMC3377601

[30] Huson DH, Bryant D (2006) Application of phylogenetic networks in evolutionary studies. Mol Biol Evol 23:254–267. 10.1093/molbev/msj030 16221896 10.1093/molbev/msj030

[31] Jain C, Rodriguez-R LM, Phillippy AM, Konstantinidis KT (2018) High throughput ANI analysis of 90K prokaryotic genomes reveals clear species boundaries. Nat Commun 9:5114. 10.1038/s41467-018-07641-9 30504855 10.1038/s41467-018-07641-9 PMC6269478

[32] Fang FC, Libby SJ, Buchmeier NA et al (1992) The alternative sigma factor katF (rpoS) regulates Salmonella virulence. Proc Natl Acad Sci U S A 89:11978–11982. 10.1073/pnas.89.24.11978 1465428 10.1073/pnas.89.24.11978 PMC50681

[33] Shah J, Desai PT, Chen D et al (2013) Preadaptation to cold stress in Salmonella enterica serovar Typhimurium increases survival during subsequent acid stress exposure. Appl Environ Microbiol 79:7281–7289. 10.1128/AEM.02621-13 24056458 10.1128/AEM.02621-13 PMC3837722

[34] Fierer J, Eckmann L, Fang F et al (1993) Expression of the Salmonella virulence plasmid gene spvB in cultured macrophages and nonphagocytic cells. Infect Immun 61:5231–5236. 10.1128/iai.61.12.5231-5236.1993 8225598 10.1128/iai.61.12.5231-5236.1993 PMC281306

[35] Pfeifer CG, Marcus SL, Steele-Mortimer O et al (1999) Salmonella Typhimurium virulence genes are induced upon bacterial invasion into phagocytic and nonphagocytic cells. Infect Immun 67:5690–5698. 10.1128/IAI.67.11.5690-5698.1999 10531217 10.1128/iai.67.11.5690-5698.1999 PMC96943

[36] Moreira CG, Weinshenker D, Sperandio V (2010) QseC mediates Salmonella enterica serovar typhimurium virulence in vitro and in vivo. Infect Immun 78:914–926. 10.1128/IAI.01038-09 20028809 10.1128/IAI.01038-09 PMC2825943

[37] Everest PH, Goossens H, Butzler JP et al (1992) Differentiated Caco-2 cells as a model for enteric invasion by Campylobacter jejuni and C. coli. J Med Microbiol 37:319–332. 10.1099/00222615-37-5-319 1433253 10.1099/00222615-37-5-319

[38] Detweiler CS, Monack DM, Brodsky IE et al (2003) virK, somA and rcsC are important for systemic Salmonella enterica serovar Typhimurium infection and cationic peptide resistance. Mol Microbiol 48:385–400. 10.1046/j.1365-2958.2003.03455.x 12675799 10.1046/j.1365-2958.2003.03455.x

[39] Almeida F, Medeiros MIC, Kich JD, Falcão JP (2016) Virulence-associated genes, antimicrobial resistance and molecular typing of Salmonella typhimurium strains isolated from swine from 2000 to 2012 in Brazil. J Appl Microbiol 120:1677–1690. 10.1111/jam.13110 26913828 10.1111/jam.13110

[40] Allam US, Gopala Krishna M, Sen M et al (2012) Acidic pH induced STM1485 gene is essential for intracellular replication of Salmonella. Virulence 3:122–135. 10.4161/viru.19029 22460643 10.4161/viru.19029 PMC3396692

[41] Ramos-Morales F (2012) Acidic pH enemy or ally for enteric bacteria? Virulence 3:103–106. 10.4161/viru.19382 22460638 10.4161/viru.19382

[42] Li M, Wang K, Tang A et al (2021) Investigation of the genes involved in the outbreaks of Escherichia coli and Salmonella spp. in the United States. Antibiotics (Basel). 10.3390/antibiotics10101274 34680854 10.3390/antibiotics10101274 PMC8532668

[43] Zhao S, Li C, Hsu CH et al (2020) Comparative genomic analysis of 450 strains of Salmonella enterica isolated from diseased animals. Genes 11:1025. 10.3390/genes11091025 32883017 10.3390/genes11091025 PMC7564550

[44] Souza SSR, Turcotte MR, Li J et al (2022) Population analysis of heavy metal and biocide resistance genes in Salmonella enterica from human clinical cases in New Hampshire, United States. Front Microbiol. 10.3389/fmicb.2022.983083 36338064 10.3389/fmicb.2022.983083 PMC9626534

[45] Tetaz TJ, Luke RK (1983) Plasmid-controlled resistance to copper in Escherichia coli. J Bacteriol 154:1263–1268. 10.1128/jb.154.3.1263-1268.1983 6343346 10.1128/jb.154.3.1263-1268.1983 PMC217599

[46] Hennaux L, Kohchtali A, Bâlon H et al (2022) Refolding and biophysical characterization of the Caulobacter crescentus copper resistance protein, PcoB: an outer membrane protein containing an intrinsically disordered domain. Biochimica et Biophysica Acta (BBA) - Biomembranes. 10.1016/j.bbamem.2022.184038 36057369 10.1016/j.bbamem.2022.184038

[47] Tan Y, Zhao K, Yang S et al (2024) Insights into antibiotic and heavy metal resistance interactions in Escherichia coli isolated from livestock manure and fertilized soil. J Environ Manage. 10.1016/j.jenvman.2023.119935 38154221 10.1016/j.jenvman.2023.119935

[48] Jiang X, Siddique A, Zhu L et al (2025) Ecological prevalence and genomic characterization of Salmonella isolated from selected poultry farms in Jiangxi province, China. Poult Sci. 10.1016/j.psj.2025.105197 40279690 10.1016/j.psj.2025.105197 PMC12434246

[49] Vilela FP, dos Prazeres Rodrigues D, Allard MW, Falcão JP (2022) Prevalence of efflux pump and heavy metal tolerance encoding genes among Salmonella enterica serovar Infantis strains from diverse sources in Brazil. PLoS One. 10.1371/journal.pone.0277979 36413564 10.1371/journal.pone.0277979 PMC9681071

[50] Argudín MA, Hoefer A, Butaye P (2019) Heavy metal resistance in bacteria from animals. Res Vet Sci 122:132–147. 10.1016/j.rvsc.2018.11.007 30502728 10.1016/j.rvsc.2018.11.007

[51] Hobman JL, Crossman LC (2015) Bacterial antimicrobial metal ion resistance. J Med Microbiol 64:471–497. 10.1099/jmm.0.023036-0 25418738 10.1099/jmm.0.023036-0

[52] Mijnendonckx K, Leys N, Mahillon J et al (2013) Antimicrobial silver: uses, toxicity and potential for resistance. BioMetals 26:609–621. 10.1007/s10534-013-9645-z 23771576 10.1007/s10534-013-9645-z

[53] Whelan KF, Colleran E, Taylor DE (1995) Phage inhibition, colicin resistance, and tellurite resistance are encoded by a single cluster of genes on the IncHI2 plasmid R478. J Bacteriol 177:5016–5027. 10.1128/jb.177.17.5016-5027.1995 7665479 10.1128/jb.177.17.5016-5027.1995 PMC177279

[54] Nguyen TTH, Kikuchi T, Tokunaga T et al (2021) Diversity of the tellurite resistance gene operon in Escherichia coli. Front Microbiol. 10.3389/fmicb.2021.681175 34122392 10.3389/fmicb.2021.681175 PMC8193136

[55] Anantharaman V, Iyer LM, Aravind L (2012) Ter-dependent stress response systems: novel pathways related to metal sensing, production of a nucleoside-like metabolite, and DNA-processing. Mol Biosyst 8:3142–3165. 10.1039/c2mb25239b 23044854 10.1039/c2mb25239b PMC4104200

[56] Muñoz-Villagrán CM, Mendez KN, Cornejo F et al (2018) Comparative genomic analysis of a new tellurite-resistant Psychrobacter strain isolated from the Antarctic Peninsula. PeerJ. 10.7717/peerj.4402 29479501 10.7717/peerj.4402 PMC5822837

[57] Turkovicova L, Smidak R, Jung G et al (2016) Proteomic analysis of the TerC interactome: novel links to tellurite resistance and pathogenicity. J Proteomics 136:167–173. 10.1016/j.jprot.2016.01.003 26778143 10.1016/j.jprot.2016.01.003

[58] Wu X, Zhan F, Zhang J et al (2022) Identification of hypervirulent Klebsiella pneumoniae carrying terW gene by MacConkey-potassium tellurite medium in the general population. Front Public Health. 10.3389/fpubh.2022.946370 36091562 10.3389/fpubh.2022.946370 PMC9448990

[59] Tran TQ, Park M, Lee JE et al (2023) Analysis of antibiotic resistance gene cassettes in a newly identified Salmonella enterica serovar Gallinarum strain in Korea. Mob DNA. 10.1186/s13100-023-00292-8 37095552 10.1186/s13100-023-00292-8 PMC10124037

[60] Mindlin S, Kholodii G, Gorlenko Z et al (2001) Mercury resistance transposons of Gram-negative environmental bacteria and their classification. Res Microbiol 152:811–822. 10.1016/S0923-2508(01)01265-7 11763242 10.1016/s0923-2508(01)01265-7

[61] Naguib MM, El-Gendy AO, Khairalla AS (2018) Microbial diversity of Mer Operon genes and their potential rules in mercury bioremediation and resistance. Open Biotechnol J 12:56–77. 10.2174/1874070701812010056

[62] Slipski CJ, Jamieson-Datzkiw TR, Zhanel GG, Bay DC (2021) Characterization of proteobacterial plasmid integron-encoded qac efflux pump sequence diversity and quaternary ammonium compound antiseptic selection in Escherichia coli grown planktonically and as biofilms. Antimicrob Agents Chemother. 10.1128/AAC.01069-21 34280018 10.1128/AAC.01069-21 PMC8448097

[63] Kula N, Lamch Ł, Futoma-Kołoch B et al (2022) The effectiveness of newly synthesized quaternary ammonium salts differing in chain length and type of counterion against priority human pathogens. Sci Rep. 10.1038/s41598-022-24760-y 36526659 10.1038/s41598-022-24760-y PMC9757636

[64] de Quadros CL, Manto L, Mistura E et al (2020) Antimicrobial and disinfectant susceptibility of Salmonella serotypes isolated from swine slaughterhouses. Curr Microbiol 77:1035–1042. 10.1007/s00284-020-01904-9 32008078 10.1007/s00284-020-01904-9

[65] Osimitz TG, Droege W (2022) Adverse outcome pathway for antimicrobial quaternary ammonium compounds. J Toxicol Environ Health 85:494–510. 10.1080/15287394.2022.2037479 10.1080/15287394.2022.2037479 35191814

[66] Arnold WA, Blum A, Branyan J et al (2023) Quaternary ammonium compounds: a chemical class of emerging concern. Environ Sci Technol 57:7645–7665. 10.1021/acs.est.2c08244 37157132 10.1021/acs.est.2c08244 PMC10210541

[67] Roldan-Henao M, Dalsgaard A, Cardona-Castro N et al (2023) Pilot study of the productivity and Salmonella seroprevalence in pigs administered organic acids. Front Vet Sci. 10.3389/fvets.2023.1123137 36937024 10.3389/fvets.2023.1123137 PMC10020582

[68] Stingelin GM, Scherer RS, Machado AC et al (2023) The use of thymol, carvacrol and sorbic acid in microencapsules to control Salmonella Heidelberg, S. Minnesota and S. Typhimurium in broilers. Front Vet Sci. 10.3389/fvets.2022.1046395 36686174 10.3389/fvets.2022.1046395 PMC9846790

[69] Bonilla KP, Vega D, Maher J et al (2023) Validation of commercial antimicrobial intervention technologies to control Salmonella on skin-on market hog carcasses and chilled pork wholesale cuts. Food Control. 10.1016/j.foodcont.2023.109829

[70] Ciríaco M, Moura-Alves M, Silva R et al (2021) Decontamination of Pig Carcasses with Organic Acids. Proceedings 70. 10.3390/foods_2020-07649

[71] Silano V, Barat Baviera JM, Bolognesi C et al (2018) Evaluation of the safety and efficacy of the organic acids lactic and acetic acids to reduce Microbiological surface contamination on pork carcasses and pork cuts. EFSA J 16. 10.2903/j.efsa.2018.5482 10.2903/j.efsa.2018.5482 PMC7009387 32625776

[72] Carvajal A, Kramer M, Argüello H (2024) Salmonella control in swine: a thoughtful discussion of the pre- and post-harvest control approaches in industrialized countries. Animals. 10.3390/ani14071035 38612274 10.3390/ani14071035 PMC11010990

[73] Gu D, Xue H, Yuan X et al (2021) Genome-Wide identification of genes involved in acid stress resistance of Salmonella derby. Genes (Basel) 12:476. 10.3390/genes 33806186 10.3390/genes12040476 PMC8065570

[74] Wessels K, Rip D, Gouws P (2021) Salmonella in chicken meat: consumption, outbreaks, characteristics, current control methods and the potential of bacteriophage use. Foods. 10.3390/foods10081742 34441520 10.3390/foods10081742 PMC8394320

[75] Karash S, Liyanage R, Qassab A et al (2017) A comprehensive assessment of the genetic determinants in Salmonella Typhimurium for resistance to hydrogen peroxide using proteogenomics. Sci Rep. 10.1038/s41598-017-17149-9 29213059 10.1038/s41598-017-17149-9 PMC5719062

[76] Maertens H, De Reu K, Meyer E et al (2019) Limited association between disinfectant use and either antibiotic or disinfectant susceptibility of Escherichia coli in both poultry and pig husbandry. BMC Vet Res. 10.1186/s12917-019-2044-0 31477099 10.1186/s12917-019-2044-0 PMC6721165

[77] Ostovan R, Pourmontaseri M, Hosseinzadeh S, Shekarforoush SS (2021) Interaction between the probiotic Bacillus subtilis and Salmonella typhimurium in Caco-2 cell culture. Iran J Microbiol 13:91–97. 10.18502/ijm.v13i1.5497 33889367 10.18502/ijm.v13i1.5497 PMC8043819

[78] Barrila J, Yang J, Crabbé A et al (2017) Three-dimensional organotypic co-culture model of intestinal epithelial cells and macrophages to study Salmonella enterica colonization patterns. NPJ Microgravity. 10.1038/s41526-017-0011-2 28649632 10.1038/s41526-017-0011-2 PMC5460263

[79] Li Q, Wang X, Xia J et al (2018) Salmonella-containing vacuole development in avian cells and characteristic of cigR in Salmonella enterica serovar Pullorum replication within macrophages. Vet Microbiol 223:65–71. 10.1016/j.vetmic.2018.07.013 30173754 10.1016/j.vetmic.2018.07.013

