# Supplementary material for: Genotypic and Phenotypic Characterization of Salmonella Typhimurium Strains Isolated from Swine in the Southern Region of Brazil
Source: Curr Microbiol. 2026 Feb 21;83(4):203. doi: 10.1007/s00284-026-04762-z (PMC12924845; doi:10.1007/s00284-026-04762-z)
Supplement: Supplementary file 1 — Supplementary Material 1 [file 284_2026_4762_MOESM1_ESM.docx]

| **Genes** | **Conditions** | **Mean absence** | **Mean presence** | **p-value** |
| --- | --- | --- | --- | --- |
| ***qacEdelta1*** | Oxidative 10 m | 30,91 | 33,09 | 0,85 |
|  | Oxidative 1 h | 7,95 | 10,01 | 0,76 |
|  | Acid 10 m | 95,72 | 97,73 | 0,62 |
|  | Acid1 h | 82,56 | 95,33 | 0,18 |
|  | Caco-2 | 63,46 | 50,00 | 0,19 |
|  | U937 | 55,77 | 62,50 | 0,43 |
| ***qacL*** | Oxidative 10 m | 32,25 | 28,94 | 0,77 |
|  | Oxidative 1 h | 7,81 | 11,03 | 0,69 |
|  | Acid 10 m | 96,02 | 97,16 | 0,79 |
|  | Acid1 h | 83,80 | 94,16 | 0,29 |
|  | Caco-2 | 64,29 | 43,75 | **0,03*** |
|  | U937 | 58,04 | 56,25 | 0,88 |
| ***pcoABCDERS*** | Oxidative10 m | 37,91 | 25,12 | 0,26 |
|  | Oxidative 1 h | 6,72 | 10,33 | 0,58 |
|  | Acid 10 m | 94,19 | 98,35 | 0,41 |
|  | Acid1 h | 78,79 | 93,42 | 0,23 |
|  | Caco-2 | 58,33 | 61,11 | 0,75 |
|  | U937 | 59,72 | 55,56 | 0,57 |
| ***silABCEFPRS*** | Oxidative 10 m | 25,12 | 37,91 | 0,26 |
|  | Oxidative 1 h | 10,33 | 6,72 | 0,58 |
|  | Acid 10 m | 98,35 | 94,19 | 0,41 |
|  | Acid1 h | 93,42 | 78,79 | 0,23 |
|  | Caco-2 | 61,11 | 58,33 | 0,75 |
|  | U937 | 55,56 | 59,72 | 0,57 |
| ***merCPRT*** | Oxidative10 m | 32,75 | 21,67 | 0,13 |
|  | Oxidative 1 h | 9,47 | 1,01 | **0,03*** |
|  | Acid 10 m | 98,36 | 79,56 | 0,53 |
|  | Acid1 h | 88,11 | 70,04 | 0,65 |
|  | Caco-2 | 56,25 | 87,50 | 0,22 |
|  | U937 | 56,25 | 68,75 | 0,24 |
| ***terDWZ*** | Oxidative10 m | 24,58 | 49,55 | 0,12 |
|  | Oxidative 1 h | 7,69 | 10,71 | 0,65 |
|  | Acid 10 m | 95,72 | 97,73 | 0,62 |
|  | Acid1 h | 84,50 | 90,28 | 0,57 |
|  | Caco-2 | 61,54 | 55,00 | 0,53 |
|  | U937 | 54,81 | 65,00 | 0,14 |

**Table S1.** Comparison of mean survival (%) between *Salmonella* Typhimurium isolates carrying or lacking stress-related genes under different phenotypic conditions. P-values were obtained using Student’s t-test.
